# Recording COVID-19 consultations: review of symptoms, risk factors, and proposed SNOMED CT terms

**DOI:** 10.3399/bjgpopen20X101125

**Published:** 2020-08-26

**Authors:** Bhautesh Dinesh Jani, Jill P Pell, Dylan McGagh, Harshana Liyanage, Dave Kelly, Simon de Lusignan, Christopher J Weatherburn, Ronnie Burns, Frank M Sullivan, Frances S Mair

**Affiliations:** 1 Clinical Senior Lecturer in General Practice and Primary Care, Institute of Health and Wellbeing, University of Glasgow, Glasgow, UK; 2 Henry Mechan Professor of Public Health, Institute of Health and Wellbeing, University of Glasgow, Glasgow, UK; 3 Medical Sciences Divison, University of Oxford, Oxford, UK; 4 Nuffield Department of Primary Care Health Sciences, University of Oxford, Oxford, UK; 5 Albasoft Limited, Centre for Health Science, Inverness, UK; 6 Professor of Primary Care and Clinical Informatics, Nuffield Department of Primary Care Health Sciences, University of Oxford, Oxford, UK; 7 Scottish Clinical Information Management in Practice, National Services Scotland, Edinburgh, UK; 8 McKenzie Burns Practice, Parkhead Health Centre, Glasgow, UK; 9 Professor of Primary Care Medicine, Medical School, University of St Andrews, St Andrews, UK; 10 Norie Miller Professor of General Practice, Institute of Health and Wellbeing, University of Glasgow, Glasgow, UK

**Keywords:** COVID-19, Systematised Nomenclature of Medicine, General practice, Primary health care, Medical Records Systems, Computerised

## Abstract

**Background:**

There is an urgent need for epidemiological research in primary care to develop risk assessment processes for patients presenting with COVID-19, but lack of a standardised approach to data collection is a significant barrier to implementation.

**Aim:**

To collate a list of relevant symptoms, assessment items, demographics, and lifestyle and health conditions associated with COVID-19, and match these data items with corresponding SNOMED CT clinical terms to support the development and implementation of consultation templates.

**Design & setting:**

Published and preprint literature for systematic reviews, meta-analyses, and clinical guidelines describing the symptoms, assessment items, demographics, and/or lifestyle and health conditions associated with COVID-19 and its complications were reviewed. Corresponding clinical concepts from SNOMED CT, a widely used structured clinical vocabulary for electronic primary care health records, were identified.

**Method:**

Guidelines and published and unpublished reviews (*N* = 61) were utilised to collate a list of relevant data items for COVID-19 consultations. The NHS Digital SNOMED CT Browser was used to identify concept and descriptive identifiers. Key implementation challenges were conceptualised through a Normalisation Process Theory (NPT) lens.

**Results:**

In total, 32 symptoms, eight demographic and lifestyle features, 25 health conditions, and 20 assessment items relevant to COVID-19 were identified, with proposed corresponding SNOMED CT concepts. These data items can be adapted into a consultation template for COVID-19. Key implementation challenges include: 1) engaging with key stakeholders to achieve ’buy in’; and 2) ensuring any template is usable within practice settings.

**Conclusion:**

Consultation templates for COVID-19 are needed to standardise data collection, facilitate research and learning, and potentially improve quality of care for COVID-19.

## How this fits in

Recording of clinical features of COVID-19 presentation in primary care is likely to be poor in the absence of a template. A rapid literature review was therefore conducted to collate a comprehensive list of symptoms, assessment items, demographics, and lifestyle and health conditions associated with COVID-19, with proposed corresponding SNOMED CT clinical terms. The evidence base around COVID-19 is rapidly evolving and this list may have to be updated in the future as new evidence becomes available. These data items can be adapted into a consultation template to standardise data collection, which in turn can facilitate research and improve quality of record keeping. In practice, there are often multiple SNOMED CT clinical terms for recording a single data item. A professional consensus will be needed among clinician users to agree on appropriate SNOMED CT clinical terms to be used in a template. Implementation and widespread use of a COVID-19 consultation template will need positive engagement with key stakeholders.

## Introduction

The COVID-19 pandemic is an unprecedented global health challenge. Understanding of the epidemiology and clinical features of COVID-19 is rapidly evolving. New evidence is constantly emerging, with articles being published or uploaded on preprint servers regularly and various public health bodies, nationally and internationally, releasing their respective guidelines.

COVID-19 consultations in primary care are further complicated by contextual factors. In the UK, most of the consultations for patients with COVID-19 related symptoms are conducted remotely.^1–3^ This usually involves more than one screen being used at the same time, which makes it challenging for GPs to accurately recall and record all relevant clinical features during their consultation with patients with suspected COVID-19 symptoms. When the consultation is in-person in the surgery, personal protective equipment (PPE) restricts normal recording patterns, and this is even worse during home visits.

Good quality epidemiological studies are urgently needed to understand COVID-19.^4^ Most of the epidemiological studies until now have been conducted in secondary care.^5^ There is an urgent need for high-quality primary care epidemiological studies for COVID-19. Recently, the use of COVID-19 specific codes have been recommended but these codes mainly cover process measures and diagnostic terms, not clinical presentation features such as symptoms and assessment.^6,7^ Previous research has suggested that only 37% of clinical problems were coded in primary care records.^8^ Underreporting of relevant clinical features for COVID-19 in primary care is likely during COVID-19 related consultations, which will be a significant hindrance for high quality primary care research. A computerised, condition-specific template has previously been shown to improve the quality of care and recording in primary care, and it could be implemented for COVID-19.^9^

The aim of this study was to collate a list of relevant symptoms, assessment items, demographics, and lifestyle and health conditions associated with COVID-19 and its complications, and match these data items with proposed examples of corresponding SNOMED CT clinical terms, which will support the development and implementation of primary care consultation templates. The clinical utility of any template developed based on this work will be to help clinicians record consultations for patients presenting with suspected COVID-19 related symptoms in primary care.

## Method

The review of clinical terms involved three distinct stages: 1) search of clinical guidelines, and published and preprint literature to collate the list of relevant clinical data items for COVID-19 consultation; 2) identify examples of corresponding SNOMED CT codes that map to the identified clinical data items; and 3) conceptualise implementation challenges through an NPT lens.

### Search for clinical data items for COVID-19

The authors searched PubMed, the preprint server medRxiv, and centre for evidence-based medicine websites for reviews using ’COVID-19‘ as a keyword on 14 May 2020. The PubMed search resulted in 963 articles, while 548 articles were found on preprint server medRxiv. The inclusion criteria were systematic reviews and meta-analyses relating to symptoms, sociodemographic and lifestyle factors, past medical history, and assessment of COVID-19 . As the focus was related to primary care assessment, reviews relating to blood tests and imaging for COVID-19 were excluded. Twenty-one systematic reviews on preprint servers and 24 published systematic reviews from PubMed were identified. The authors searched for COVID-19 related guidelines on Public Health England, Health Protection Scotland, Public Health Wales, British Medical Journal (BMJ), Royal College of General Practitioners (RCGP), National Institute for Health and Care Excellence (NICE), and World Health Organization (WHO) websites. Sixteen clinical guidelines were considered for collating the list of COVID-19 consultation data items described above.

### Search for corresponding clinical terms for COVID-19

SNOMED CT is the most comprehensive structured clinical terminology in use around the world and can be used in electronic health records (http://www.snomed.org/snomed-ct/why-snomed-ct). This international clinical terminology has been implemented across various health and care settings, including primary and secondary care. Principles outlined in *Data Quality Guidance: Transitioning from Read to SNOMED CT* by NHS Digital were used for choosing examples of proposed SNOMED CT clinical terms.^10^ First, the authors referred to appropriate SNOMED hierarchies, for example, ‘disorders’ or ‘situation’ for recording health conditions, and ‘finding’ or ‘observable entity’ for recording symptoms and signs. Only SNOMED CT codes that had equivalent Read codes were considered, as Read codes are still in use in general practice in Scotland. Codes that were regarded as ‘inactive’ and no longer in use were also excluded. SNOMED CT UK edition (version 20200415) was interrogated by the NHS Digital web browser to search for terms and identifiers for each of the clinical data items identified from the previous step. Additionally, the SNOMED CT code identified was matched to the corresponding Read version 2 codes using NHS Digital browser.^11^ 

### Conceptualising implementation of consultation template

GP consultation templates are often viewed as less patient-centred and potentially disruptive to communication.^12^ The majority of existing consultation templates are used for management of long-term conditions; however, they have also been used for recording management of acute infections.^13^ Widespread adaptation of a template in a short timeframe can provide significant implementation challenges. An NPT lens was used to conceptualise these challenges and corresponding key strategies to deal with those challenges.^14^

## Results

### Clinical data items and SNOMED CT codes

In total, searches identified 32 symptoms associated with COVID-19 presentation from 10 guidelines,^1,3,15–22^ 13 published reviews,^23–35^ and 12 preprint reviews.^36–47^ See Table 1 for the list of symptoms and proposed corresponding SNOMED CT codes. The symptoms of COVID-19 were heterogenous in nature and included flu-like; respiratory; gastrointestinal; central nervous system; ear, nose, and throat; and eye symptoms reported in the literature.

**Table 1.** Symptoms and assessment items relevant for COVID-19 primary care consultation with respective SNOMED CT concept identifiers

Symptoms (with respective SNOMED CT concept identifiers)	Primary care assessment (with respective SNOMED CT concept identifiers)
1. Date of onset of symptoms SCTID: 5201910000001032. H/O fever SCTID: 866621000000103 (common)3. C/O cough SCTID: 272039006 (common)4. Productive cough SCTID: 28743005 (common)5. Fatigue-symptom (finding) SCTID: 272060000 (common)6. Myalgia/muscle pain SCTID: 68962001 (common)7. Dyspnoea SCTID: 267036007 (common)8. Has a sore throat SCTID: 162388002 (common)9. C/O anosmia SCTID: 272028008 (common)10. C/O loss of taste sense SCTID: 272041007 (common)11. Bloodstained sputum SCTID 6128100512. Chest pain SCTID: 2985700913. Joint pain SCTID: 5767600214. C/O shivering SCTID: 16185500315. Dizziness SCTID: 40464000316. C/O a headache SCTID: 27202700317. H/O disturbance of consciousness SCTID: 9111100000010818. Convulsion SCTID: 9117500019. Syncope symptom SCTID: 27203000520. Nausea SCTID: 422587007121. C/O vomiting SCTID: 27204400422. Loss of appetite SCTID: 7989000623. Diarrhoea SCTID: 6231500824. Abdominal pain SCTID: 2152200125. Gastrointestinal haemorrhage SCTID: 7447400326. Nasal symptoms SCTID: 24930700327. Rhinorrhoea SCTID: 6453100328. C/O nasal congestion SCTID: 27203400129. Respiratory symptom SCTID: 16192000130. Red eye SCTID: 70363000331. C/O a rash SCTID: 16241500832. Palpitations SCTID: 80313002	1. Rockwood Clinical Frailty Scale SCTID: 4454140072. Dyspnoea on exertion SCTID: 608450063. Breathless at rest (finding) SCTID: 1619410074. Ability to perform activities of everyday life SCTID: 2845450015. Unable to climb stairs SCTID: 1652470066. Normal fluid intake SCTID: 1618430097. Not taking fluids (finding) SCTID: 1618450028. Oliguria SCTID: 831280099. Anuria (finding) SCTID: 247200210. Unable to stand (finding) SCTID: 16190300011. C/O cold extremities SCTID: 16199600112. Blue lips (finding) SCTID: 16274300013. Mentally alert SCTID: 24823400814. O/E confused SCTID: 16270200015. O/E disorientated SCTID: 163608000Vital Observations (if available)16. O/E temperature (observable entity) SCTID: 30964600817. O/E pulse rate SCTID: 16298600718. Peripheral oxygen saturation SCTID: 43131400419. O/E blood pressure reading SCTID: 16302000720. O/E respiratory rate SCTID: 162913005

C/O= complains of. H/O = history of. O/E = on examination. SCTID = SNOMED CT Identifier

The clinical domain of primary care assessment for COVID-19 had the least number of supporting reviews (two preprint reviews)^39,48^ and the majority of the data items included were on the basis of clinical guidelines,^1–3,16–20,49,50^ which in turn were based on expert opinions (Table 1). The data items included for COVID-19 assessment were based on data items validated for general practice consultations, but not specifically for COVID-19 (for example, telephone consultation for suspected sepsis^49^ and use of the NEWS2 tool^1^).

Demographic factors (age, sex, and ethnicity) were reported to be relevant for COVID-19 susceptibility and complications by two guidelines,^15,20^ 10 published reviews,^5,25,26,28,30,33,51–54^ and seven preprint reviews.^37,43,55–59^ Lifestyle factors (working, smoking, and body mass index) were reported to be relevant for COVID-19 related assessment by two guidelines,^15,20^ one published review,^51^ and seven preprint reviews.^37,43,55–57,59,60^ Individuals working in the health profession were regarded as high risk by one guideline.^20^ See Table 2 for the list of relevant demographics and lifestyle factors, and examples of corresponding SNOMED CT codes (apart from clinical terms for age and sex, which are usually recorded in all primary care records).

**Table 2.** Demographic, lifestyle, and existing health conditions relevant for COVID-19 primary care consultations

Lifestyle and demographics (with respective SNOMED CT concept identifiers)	Past medial history (PMH) relevant for COVID-19 primary care consultation (with respective SNOMED CT concept identifiers)
1. Age^a^2. Sex^a^3. Ethnicity/related nationality data (observable entity) SCTID: 1860340074. Health profession SCTID: 2233660095. Current smoker SCTID: 771760026. Ex-smoker SCTID: 85170067. Non-smoker SCTID: 83920008. Finding of body mass index SCTID: 301331008	1. High-risk category for developing complication from COVID-19 infection SCTID: 13005610000001072. H/O diabetes mellitus SCTID: 1614450093. H/O hypertension SCTID: 1615010074. Ischaemic heart disease SCTID: 4145450085. H/O heart failure SCTID: 1615050036. Rheumatic heart disease SCTID: 236850007. H/O atrial fibrillation SCTID: 3124420058. Cardiac arrhythmia SCTID: 6982470079. Congenital heart disease SCTID: 1321300910. Pulmonary heart disease SCTID: 27409600011. H/O asthma SCTID: 16152700712. History of chronic obstructive pulmonary disease SCTID: 27047300113. Pulmonary emphysema SCTID: 8743300114. H/O bronchiectasis SCTID: 93943100000010815. Cystic fibrosis SCTID: 19090500816. Pulmonary fibrosis SCTID: 5161500117. Chronic kidney disease SCTID: 70904400418. Chronic liver disease SCTID: 32838300119. H/O stroke SCTID: 27552600620. H/O epilepsy SCTID: 16148000821. H/O dementia SCTID: 16146500222. Multiple sclerosis SCTID: 2470000723. Motor neurone disease SCTID: 3734000024. Patient immunocompromised SCTID: 370388006*25*. Pregnant SCTID: 77386006

^a^Information on age and sex is usually documented on all electronic primary care records and is not needed in a consultation template. H/O = history of. SCTID = SNOMED CT Identifier.

In total, 25 health conditions were identified with COVID-19 vulnerability and/or complications, reported by two guidelines,^15,20^ 16 published reviews^5,25–28,30–33,54,61–66^ and 10 preprint reviews.^37,39,42,45,47,57,59,60,67,68^ The list of conditions included the ’high-risk‘ or ’extremely vulnerable from COVID-19’ criteria based on guidelines issued by Public Health England and Health Protection Scotland.^69,70^ The list included a breadth of conditions ranging from cardiovascular, respiratory, immunosuppressed conditions, and previous cancer (see Table 2 for the list of medical conditions and the proposed examples of corresponding SNOMED CT codes). The mapping Read version 2 codes for the SNOMED CT codes identified are listed in Supplementary file Tables S1-4.

### Implementation challenges and solutions

**Figure 1.**
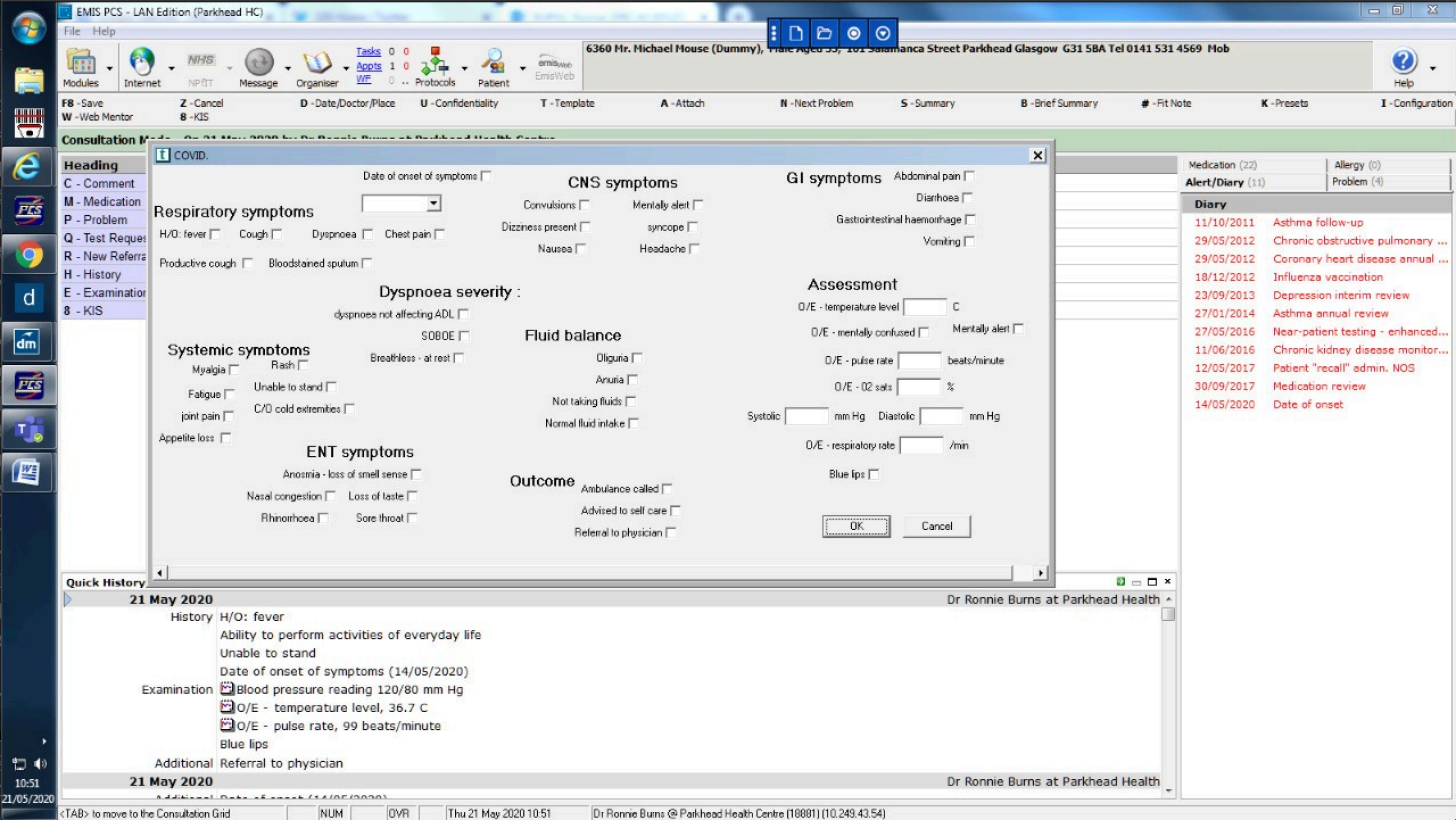
A consultation template prototype in EMIS with symptoms and assessment items for recording COVID-19 consultations in primary care

There is an extensive literature regarding barriers and facilitators to digital health implementation.^71^ This literature has demonstrated the importance of considering implementation issues whenever introducing any new digital health system, including those relating to electronic medical record systems.^72^ While electronic medical record systems are fully integrated within general practice in the UK, changes to functionality or uptake and utilisation of new templates within electronic medical record systems may not become widespread unless potential barriers or facilitators to use are considered.^73^ NPT is a theory of implementation that has been used extensively over the last decade to understand how new service innovations, particularly digital health innovations, can become embedded and implemented, or not, as part of routine practice.^14^ It suggests the need to consider issues relating to: coherence, how people make sense of a new way of working; cognitive participation, the engagement work required to get people to ’buy into‘ adopting a new way of working; collective action, the work of operationalising a new digital tool; and reflexive monitoring, the work of appraising, judging, or adapting a new digital innovation. There is evidence that considering and addressing such issues at the start of any new digital intervention will enhance the likelihood of routine embedding as part of normal practice. Table 3 outlines key issues that should be addressed in order to enhance uptake and utilisation of any new COVID-19 template. As a proof of concept, a COVID-19 consultation template prototype using data items relevant for symptoms and assessment was developed at a practice level in EMIS (Figure 1). Such consultation template prototypes may have to be modified after operationalisation, based on user feedback.

**Table 3.** Key Normalisation Process Theory-informed implementation issues to consider when introducing a new COVID-19 template

Coherence	Ensuring that potential users are aware of the new template and that they understand the rationale for trying to promote utilisation with every COVID-19 patient.
Cognitive participation	Engagement with users will be key to ensure they will invest time using the template.Ensuring that the use of the template should assist primary care clinicians in recording relevant clinical items rather than act as a hindrance or source of additional work.Ensuring users are clear that the items included in the template are evidence based and that it is clinically worthwhile to record the data items within the template.Champions within the GP workforce can be valuable to promote uptake.
Collective action	Any template needs to be easy to navigate and use. A template that is too time-consuming to complete is unlikely to achieve widespread utilisation. Consequently, any template needs to be embedded in the currently used electronic medical record system.
Reflexive monitoring	Providing feedback to users regarding data collected from the template will enable them to see the potential benefits and learning that can be gained through use of the template. Providing a mechanism to allow iterative feedback so that the template can be improved in line with user comments would also be important.

## Discussion

The authors identified 32 symptoms, 20 assessment items, eight demographic and lifestyle features, and 25 health conditions relevant for recording in COVID-19 primary care consultations. The supporting evidence base for these data items comprised of 16 clinical guidelines, 24 published, and 21 unpublished systematic reviews. The authors propose examples of corresponding clinical terms for all clinical data items from the SNOMED CT browser, which can be used to develop consultation templates for different primary care IT system providers. The authors also discuss the likely implementation challenges and potential solutions for large scale roll out of a COVID-19 consultation template. The contents of any COVID-19 consultation template may have to be adapted in future as knowledge and understanding evolves.

### Strengths and limitations

This is the first study to collate a list of relevant clinical items and corresponding clinical terms for COVID-19 consultation. The search for preprint servers enabled the authors to include the most up-to-date evidence, with the caveat that these platforms publish rapid non-peer reviewed literature. This study also outlines an implementation blueprint using a well-known theoretical framework for complex interventions.

There are several limitations. First, the authors did not employ a systematic search strategy for the literature review, and did not conduct a quality appraisal of the included studies as these were not the objectives of this study. The study objective was to facilitate standardised data collection, which in turn can lead to high quality research and strengthen the evidence base. Several data items were included from preprint studies that have not undergone the scrutiny of peer review, and future evidence may suggest that recording of these data items is not necessary for risk assessment in primary care.

### Comparison with existing literature

Implementation of a COVID-19 consultation template is likely to face similar challenges to those observed with implementation of other general practice consultation templates. The previous positive experiences with the use of a template have included providing a structured consultation and being more comprehensive, while the use of a template has also been perceived as less patient-centred and more focused on a biomedical agenda.^12,74,75^ The uptake of a template in clinical practice is not likely to be universal; clinicians may need prompting and the template may require reinforcing to improve utilisation.^13^ There have been very few primary care-based studies of COVID-19, and these studies have not been able to report on symptoms or assessment due to inconsistencies in recording of symptoms in primary care.^76,77^

### Implications for research and practice

The list of clinical terms can lead to the development of COVID-19 consultation templates, which in turn will help with standardised data collection and high quality research. A consultation template can potentially help with quality of clinical care and record keeping by facilitating comprehensive coverage of various clinical domains relevant to COVID-19.^78^ The use of a consultation template along with coding of other process measures included in the recently published ontology^79^  and SNOMED CT codes on COVID-19 released by NHS Digital^80^ can also help with disease surveillance. Several data items related to demographics, lifestyle, and past medical history may already be recorded in patient’s existing primary care records. These items are still included in the template as they will be relevant in out-of-hours settings where this information may not be readily available. The authors acknowledge that the codes presented are only examples for recording the relevant clinical data items. In practice, there are often multiple ways of recording a single data item and previous research has suggested evidence of considerable heterogeneity in SNOMED CT coding.^81,82^ A professional consensus exercise will be needed among clinician users to agree on appropriate SNOMED CT clinical terms. The use of a COVID-19 consultation template has the potential to offer consistency in data collection, albeit with some variations in codes used to record clinical data items.

## Notes

SdeL is Director of the Royal College of General Practitioners, Research and Surveillance Centre (English national surveillance system). SdeL has received commercial funding for investigator led research, and belongs to advisory boards, but not related to COVID-19 and all through his university.
